# Effects of the PbBr_2_:PbI_2_ Molar Ratio on the Formation of Lead Halide Thin Films, and the Ratio’s Application for High Performance and Wide Bandgap Solar Cells

**DOI:** 10.3390/ma15030837

**Published:** 2022-01-22

**Authors:** Md. Abdul Kuddus Sheikh, Son Singh, Rahim Abdur, Sung-Min Lee, Jae-Hun Kim, Ho-Seok Nam, Hyunseung Lee, Jaegab Lee

**Affiliations:** 1 School of Advanced Materials Engineering, Kookmin University, Seoul 02707, Korea; abdulkuddus@kookmin.ac.kr (M.A.K.S.); ssingh@kookmin.ac.kr (S.S.); jonyjosh143@gmail.com (R.A.); sungminlee@kookmin.ac.kr (S.-M.L.); jaehunkim@kookmin.ac.kr (J.-H.K.); hsnam@kookmin.ac.kr (H.-S.N.); 2 Department of Fashion Industry, Incheon National University, Incheon 22012, Korea; srwalpha@inu.ac.kr

**Keywords:** mixed lead halide, surface morphology, microstructure, solar cells, performance, stability

## Abstract

We investigate the effects of the molar ratio (x) of PbBr_2_ on the phases, microstructure, surface morphology, optical properties, and structural defects of mixed lead halides PbI_2(1−x)_Br_2x_ for use in solar cell devices. Results indicate that as x increased to 0.3, the surface morphology continued to improve, accompanied by the growth of PbI_2_ grains. This resulted in lead halide films with a very smooth and continuous morphology, including large grains when the film was formed at x = 0.3. In addition, the microstructure changed from (001)-oriented pure PbI_2_ to a highly (001)-oriented β (PbI_2_-rich) phase. The plausible mechanism for the enhanced morphology of the lead halide films by the addition of PbBr_2_ is proposed based on the growth of a Br-saturated lead iodide solid solution. Furthermore, iodine vacancies, identified by X-ray photoelectron spectroscopy, decreased as the ratio of PbBr_2_ increased. Finally, an electrical analysis of the solar cells was performed by using a PN heterojunction model, revealing that structural defects, such as iodine vacancies and grain boundaries, are the main contributors to the degradation of the performance of pure PbI_2_-based solar cells (including high leakage, low stability, and high hysteresis), which was significantly improved by the addition of PbBr_2_. The solar cell fabricated at x = 0.3 in air showed excellent stability and performance. The device lost merely 20% of the initial efficiency of 4.11% after 1500 h without encapsulation. This may be due to the dense microstructure and the reduced structural defects of lead halides formed at x = 0.3.

## 1. Introduction

PbI_2_ is a highly photoconductive semiconductor with a direct bandgap, high absorption coefficient, and good flexibility [1,2,3,4,5,6]. PbI_2_ is frequently used to fabricate organic-inorganic halide perovskite solar cells [7], and as γ-ray or X-ray detectors, photovoltaics, photodetectors, and lasing devices [5,8,9,10,11,12,13]. In addition, PbI_2_ is a layered material with a repeating unit of a hexagonal close-packed layer of lead ions sandwiched between two layers of iodide ions. Each of these PbI_2_ layers interacts weakly with adjacent layers, giving rise to different stacking patterns, and thus leading to various polytypes in single crystals. PbI_2_ single crystals have commonly been fabricated by the Bridgeman-based technique, vapor growth, and the hot wall technique [1,9,11,13,14,15,16,17,18], while polycrystalline thin films are formed by vacuum evaporation or the spin/spray coating of PbI_2_ solutions [19,20,21,22,23,24]. Among these techniques, the deposition of PbI_2_ polycrystalline layers from a solution is very simple, reliable, and economical. However, it is difficult to grow high quality PbI_2_ films, partly due to the decomposition of PbI_2_ during the crystal growth at high temperatures, and partly due to the weak Van Der Waals (VDW) bonding between the layers that leads to the formation of PbI_2_ slabs with interlayer voids and defective packing structures, resulting in very rough surface morphologies [2,5,23,25].

In this study, we employ a mixed solution consisting of PbI_2_ and PbBr_2_ to improve the morphology and microstructures of lead halide films. As reported in the preparation of the photoactive layer of perovskite-based solar cells by employing mixed-halide elements [26,27,28,29], adding PbBr_2_ to a PbI_2_ solution can have significant impacts on the microstructure and growth of PbI_2_, depending on the molar ratio (x) of PbBr_2_ to PbI_2_. For example, Br ions dissolved in PbI_2_ structures modify the interlayer interaction of the sandwiches, affecting the growth of PbI_2_ in the c-direction [30,31]. Moreover, the addition of PbBr_2_ above the solubility limit (approximately 5 mol.%) may produce mixtures of two phases, such as a β-solid solution (solid solution of ~5 mol.% PbBr_2_ in PbI_2_ matrix) with intermetallic compound PbBr_1.2_I_0.8_ [32]. Therefore, the change in phases and morphological features of PbI_2_ by the addition of PbBr_2_ were investigated by X-ray diffraction (XRD) and scanning electron microscopy (SEM). In addition, X-ray photoelectron spectroscopy (XPS) was used to analyze the elements, compounds, and iodine vacancies of the lead halide films, while UV-visible spectroscopy was used to analyze the optical properties. Combined with these analyses, the quasi-binary phase diagram of PbI_2_-PbBr_2_ was used to understand the compositional effects on the microstructural and morphological development of lead halide, the mechanism of which is proposed in this paper. Finally, a solar cell was fabricated based on the mixed lead halides to evaluate the film quality in terms of performance, hysteresis, and stability, revealing the relationship between the structural defects and leakage currents (shunt current and capacitive current). The addition of PbBr_2_ decreased the defects and led to the excellent performance of highly stable solar cells fabricated at the molar ratio of 0.3.

## 2. Experimental Setup and Methods

Device fabrication: Fluorine-doped tin oxide (FTO) coated glass substrates (7 Ω/sq, Sigma-Aldrich, Darmstadt, Germany) were partially etched using a chemical treatment with Zn powder and hydrochloric acid (2 M). Each substrate was cleaned ultrasonically in deionized (DI) water, acetone, and ethanol for 10 min, and then dried with N_2_ gas. Finally, the substrates were treated in an ultraviolet (UV)-ozone treatment for 30 min. A compact titanium dioxide (c-TiO_2_) layer was deposited on the FTO glass by spin-coating with a titanium diisopropoxide bis(acetylacetonate) solution (0.15 M in 1-butanol, Sigma-Aldrich, Darmstadt, Germany) at 500 rpm for 5 s and 3000 rpm for 30 s, and then dried at 100 °C for 10 min, followed by baking at 500 °C for 1 h in air. The mesoporous TiO_2_ film was deposited by spin-coating a 20 nm sized TiO_2_ paste (diluted in ethanol with a weight ratio of 1:3.5, Sigma-Aldrich, Darmstadt, Germany) at 500 rpm for 5 s and 3000 rpm for 30 s, dried at 100 °C for 10 min, and then baked at 500 °C for 1 h in air. Different contents of mixed lead halide precursor solutions were prepared to make the 1.0 M lead halide precursor solution: (A) 0.461 g (1.0 mmol) PbI_2_ powder, (B) 0.4159 g (0.9 mmol) PbI_2_ and 0.0367 g (0.1 mmol) PbBr_2_, (C) 0.3227 g (0.7 mmol) PbI_2_ and 0.1101 g (0.3 mmol) PbBr_2_, (D) 0.2305 g (0.5 mmol) PbI_2_ and 0.1835 g (0.5 mmol) PbBr_2_, and (E) 0.1383 g (0.3 mmol) PbI_2_ and 0.2569 g (0.7 mmol) PbBr_2_ were dissolved in 1 mL of N, N-dimethylformamide (DMF, 99.8%). All lead halide precursors and DMF were purchased from Sigma-Aldrich in Darmstadt, Germany. These solutions were stirred at 80 °C for 30 min in water bath. The lead halide solution was spin-coated at 3000 rpm for 30 s on the substrates in air at room temperature. The samples were annealed at 100 °C for 10 min on a hot plate in air. After that, the hole transport layer (HTL) was deposited by spin-coating a chlorobenzene solution (1 mL) containing 72.3 mg (2,2′,7,7′-tetrakis(*N*,*N*′-di-p methoxyphenylamine)-9,9-spirobifluorene) (spiro-OMeTAD), 28.8 μL of 4-tertbutyl pyridine, and 17.5 μL of a stock solution of 520 mg/mL lithium bis(trifluoromethyl sulphonyl) imide in acetonitrile (520 mg/mL) at 3000 rpm for 30 s. All the processes were performed in a clean room maintained at the temperature of 28–30 °C and relative humidity of 25–30%. Finally, 80-nm thick Au was thermally evaporated with a shadow mask under vacuum at 2 × 10^−6^ torr to act as the cathode.

Measurements and characterization: Devices with an active area of 0.3 cm × 0.3 cm were characterized in air under an Air Mass 1.5 Global (AM 1.5 G) solar simulator with an irradiation intensity of 100 mW/cm^2^. The simulator was calibrated using a standard crystalline silicon solar cell (Oriel, CA, USA). I-V curves were obtained by sweeping between −0.1 V and 1.4 V with a scan rate of 10 mV/s, whereas dark I-V curves by sweeping between −1 V and 1.4 V with a scan rate of 20 mV/s. All the measurements of the solar cells were performed under ambient conditions (relative humidity of 25–30%, temperature between 28–30 °C) without encapsulation.

The optical absorbance of the lead halide films was measured by UV-visible spectrometry (UV-3150, Shimadzu, Kyoto, Japan). The XRD patterns of the lead halide films were measured using Cu Kα radiation (λ = 0.154056 nm, 40 kV, and 200 mA, Rigaku, Tokyo, Japan). A scanning rate of 5°/min was applied to record the pattern in the range of 3–60°. The morphological features were examined by field emission scanning electron microscopy (FE-SEM, JEOL-7401F) and atomic force microscopy (AFM, Seiko, Tokyo, Japan).

The X-ray photoelectron spectroscopy of the lead halide films was performed using a K-alpha XPS system (Thermo VG, British Virgin Islands, UK). The XPS spectrum of the lead halide films was measured using mono Al X-ray sources (Al Kα line: 1486.6 eV) with a sampling area of 400 µm in diameter. A survey scan was performed using a pass energy of 200 eV and a step size of 1 eV. The vacuum level was maintained at 3.6 × 10^−9^ torr with a base pressure of 2.2 × 10^−9^ torr.

## 3. Results and Discussion

### 3.1. Formation of Mixed Lead Halides PbI_2(1−x)_Br_2x_ at Various Molar Ratio(x) of PbBr_2_

We investigated the effects of the molar ratio (x) of PbBr_2_ on the crystal structure, the preferred orientation, absorption spectra, and the morphology of a lead halide layer formed on the meso-TiO_2_ substrate.

**Figure 1:**
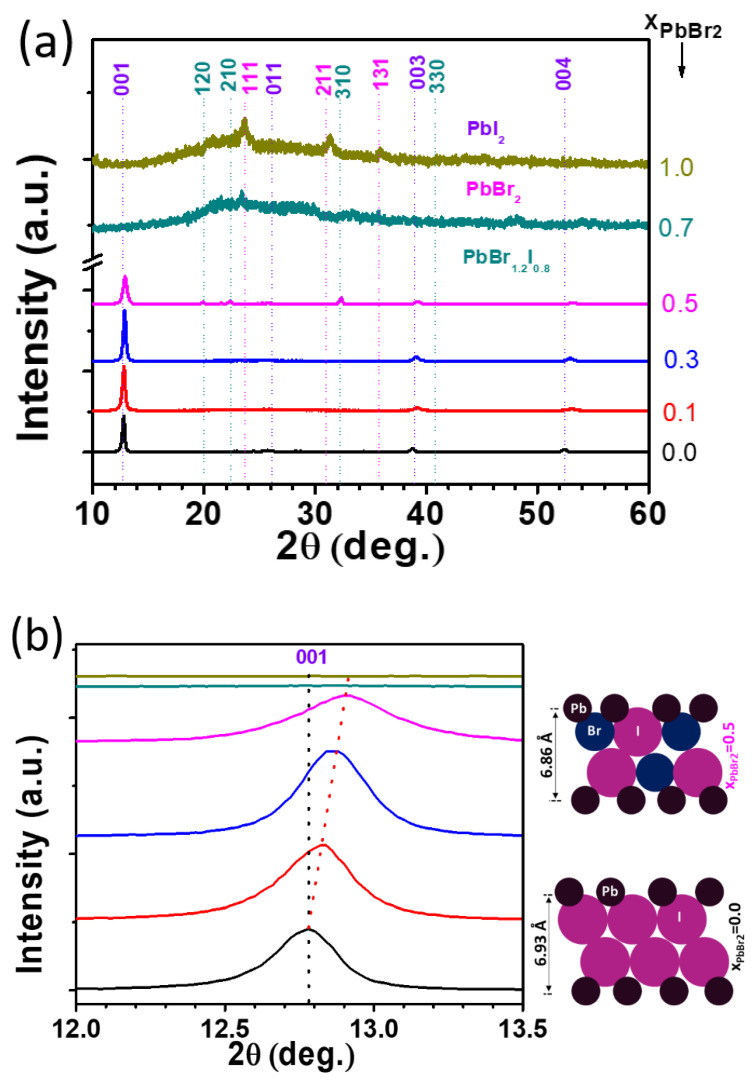
XRD patterns of (**a**) lead halide films deposited on a mesoporous TiO_2_ at various mole fractions of PbBr_2_ to PbI_2_; (**b**) the peak shift of the (001) plane of PbI_2_ to a higher 2θ with the increase in the ratio x and the schematic indicating the decreased distance between PbI_2_ layers due to the replacement of I ions by Br ions.

Figure 1a shows the XRD patterns of the lead halide films deposited on a meso-TiO_2_ layer using the mixed lead halide precursor with different molar ratios (x). For comparison, a pure PbI_2_ film was formed, and it exhibited a hexagonal structure that was oriented along the <001> direction, as shown in Figure 1. In the case of the lead halides, as the x value increases from 0.0 to 0.1 to 0.3, the intensity of the PbI_2_ (001) peak continues to increase, and the grain sizes also increases. It is also noted that the peak corresponding to the (001) plane shifts to the right with the addition of PbBr_2_, as shown in Figure 1b, implying that shrinkage of the lattice occurs in the <001> direction. This may be the result of the inclusion of Br ion, whose size is smaller than that of I ion, into I sites within a solubility limit, approximately 5% of PbBr_2_ in PbI_2_ [32]. Additionally, by increasing the PbBr_2_ concentration above 5%, which is the solid solubility, the peak continues to shift toward the right, representing a continual decrease in the c-value of the PbI_2_ unit cell along the c-axis. According to the quasi-binary phase diagram [32], two phases consisting of the β phase and intermetallic compound PbBr_1.2_I_0.8_ are formed at the molar ratio of 0.05~0.5. However, only β (PbI_2_-rich) single phase and no intermetallic compound was detected by XRD at the ratio of both 0.1 and 0.3. This implies that the Br-supersaturated PbI_2_ solid solutions are formed at x = 0.1 and 0.3 by the spin-coating of the lead halide solutions.

Furthermore, the grains of β phase continue to grow when x increases from x = 0 to 0.1 to 0.3, and then it decreases at the ratio x = 0.5, where the two phases, the β phase and PbBr_1.2_I_0.8_, are formed, as shown in Figure 1a. The dashed lines indicate the weak intensity peaks attributed to PbBr_1.2_I_0.8_. The peaks at 20.48° and 22.38° can be assigned to the (120) and (210) planes of PbBr_1.2_I_0.8_, respectively. In addition, a further increase in the ratio to x = 0.7 produced a mixture of PbBr_1.2_I_0.8_ and PbBr_2_, as shown in Figure 1b, which is consistent with the phases predicted by the phase diagram [32]. Very low intensities of the peaks of the two phases are observed, indicating that the phases are made of very fine particles.

**Figure 2:**
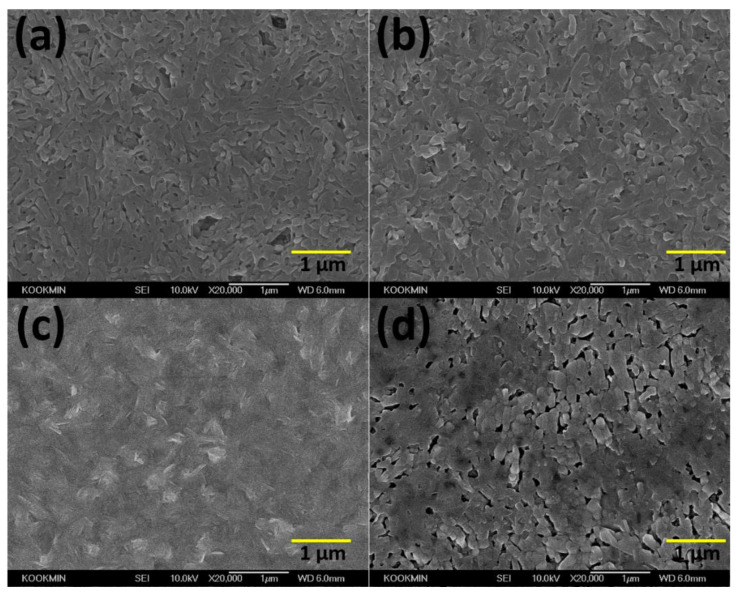
Surface morphology of lead halide films deposited on a meso-TiO_2_ substrate at x = (**a**) 0; (**b**) 0.1; (**c**) 0.3; and (**d**) 0.5.

Figure 2 shows the surface morphologies of the lead halide films deposited on meso-TiO_2_ substrates using a pure PbI_2_ solution and mixed lead halide solution, respectively. The pure PbI_2_ films show elongated-nanoslab features with relatively large voids in Figure 2a. The addition of the mole ratio x = 0.1 of PbBr_2_ decreases the size of the voids, leading to improved surface morphology. As x increases to 0.3, the surface voids are significantly decreased, leading to a smooth surface morphology. However, at the mole fraction x = 0.5, a rough surface morphology was obtained with the increase in voids, possibly due to the different characteristics of the two phases, such as nanoslab PbI_2_ and fine-grained PbBr_1.2_I_0.8_. According to the phase diagram of the PbI_2_-PbBr_2_, a eutectic product consisting of an intimate mixture of β phase and PbBr_1.2_I_0.8_ can be produced at the eutectic composition x = 0.5. The two phases in eutectics characteristically form as an alternative structure, where the fine particles of intermetallic compound may form adjacent to the elongated β phase. As a result, the morphology may be characterized mainly by the elongated-nanoslab PbI_2_ rather than the fine-grained PbBr_1.2_I_0.8_. Moreover, the growth of β phase of the eutectic structure can be decreased by fine-grained PbBr_1.2_I_0.8_ for the molar ratio x = 0.5, which is in contrast with the enhanced grain growth of single β phase by the addition of PbBr_2_ for both x = 0.1 and x = 0.3. In addition to the morphological change with x, the thickness of lead halide layers coated on meso-TiO_2_ (265 nm) measured by the cross-sectional SEM decreases from 280 nm to 210 nm to 200 nm to 190 nm as x increases from 0 to 0.1 to 0.3 to 0.5.

**Figure 3:**
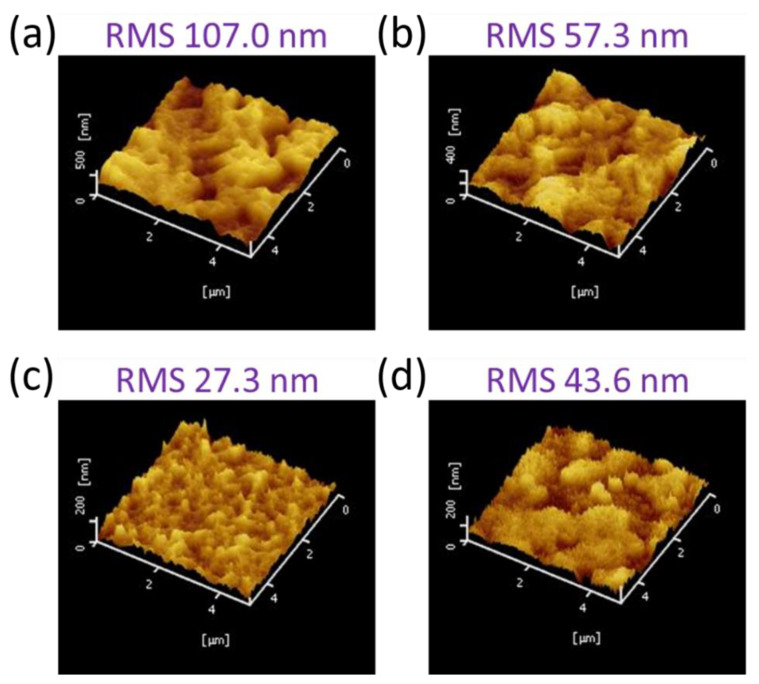
AFM images and RMS of lead halide films deposited at x = (**a**) 0; (**b**) 0.1; (**c**) 0.3; and (**d**) 0.5.

Figure 3 shows AFM images of the lead halide films deposited on meso-TiO_2_ substrates. The pure PbI_2_ films show a very rough surface with a root mean square (RMS) of 107.0 nm, as shown in Figure 3a. As the molar ratio x of PbBr_2_ increases to 0.1, the RMS decreases to 57.3 nm. A further increase in the ratio x to x = 0.3 significantly decreases the RMS to 27.3 nm. When the ratio x = 0.5, the RMS increases to 43.6 nm. The SEM images of the samples are consistent with the AFM images of the lead halide films deposited on meso-TiO_2_ substrate using a pure PbI_2_ solution and a mixed PbI_2_-PbBr_2_ solution.

X-ray photoelectron spectroscopy was used to analyze the variation in the concentration ratio of pure PbI_2_ to Br-included lead halide, as a function of the molar ratio.

**Figure 4:**
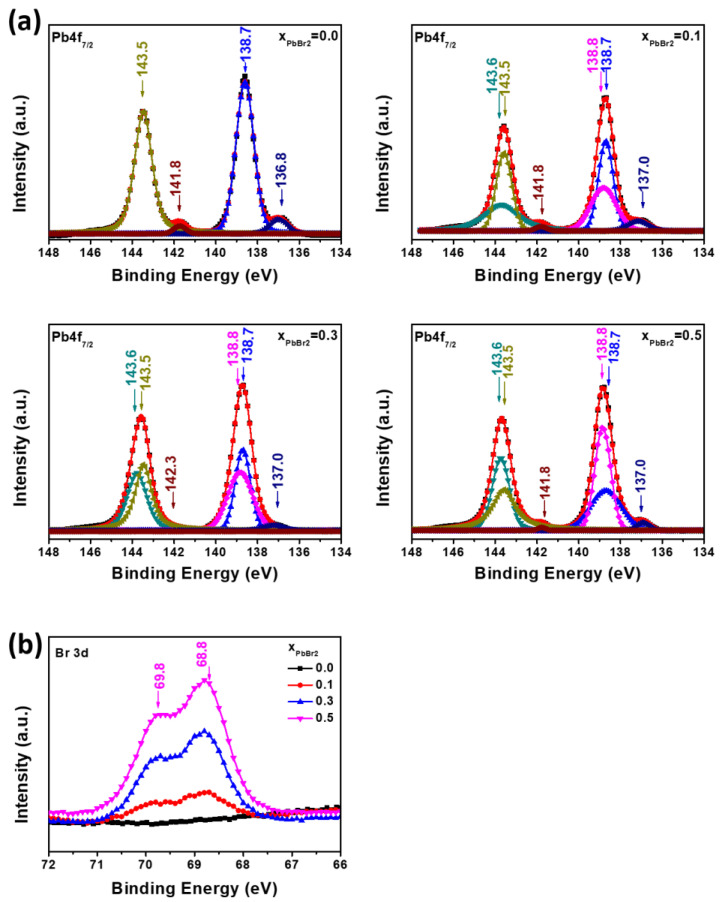
(**a**) XPS of Pb 4f spectra were de-convoluted into three peaks at 138.7, 138.6, and 137.0 eV, which are assigned to Pb 4f_7/2_ core-levels of PbI_2_, PbBr_2_, and Pb atom in the lead halide films fabricated at x = 0, 0.1, 0.3, and 0.5; (**b**) XPS Br 3d core-level spectra of lead halide films fabricated at x = 0.0, 0.1, 0.3, and 0.5.

Figure 4a shows the XPS Pb 4f spectra associated with the Pb atoms, whereas Figure 4b present the spectra associated with Br atoms [32,33,34]. The Pb core-level spectra measured at different molar ratios were de-convoluted into three peaks in Figure 4a at (138.8, 138.7, and 137) eV, which can be assigned to the Pb 4f_7/2_ core-levels in the PbI_2_, PbBr_2_, and Pb atom chemical states, respectively. As the molar ratio increases, the PbBr_2_:PbI_2_ ratio increases accordingly. In addition, Pb atoms are detected, indicating the presence of vacancies in the films because lead atoms in the charge state Pb^0^ are produced by the formation of halide vacancies around the Pb-centered polyhedra [34]. It is also noted that increasing the ratio of PbBr_2_ to PbI_2_ significantly reduces the contents of Pb^0^, implying that the addition of PbBr_2_ effectively decreases defects such as iodide vacancies. The peaks in Figure 4b at 68.8 eV are associated with Br 3d in PbBr_2_, showing that as the PbBr_2_ ratio increases from x = 0 to 0.1 to 0.3, the content of Br dissolved in PbI_2_ increases. This is consistent with Figure 4a.

**Figure 5:**
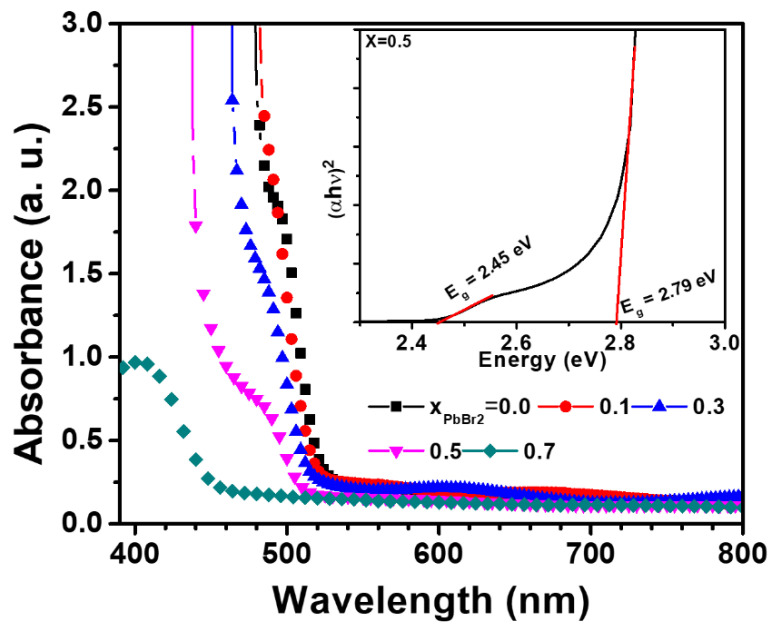
Absorbance of lead halide films deposited on glass substrates at x = 0, 0.1, 0.3, 0.5, and 0.7, respectively. The inset shows the Tauc plot of the lead halide coated at x = 0.5, revealing the optical bandgaps for PbI_2_ (2.48 eV) and PbBr_1.2_I_0.8_ (2.79 eV), respectively.

Figure 5 shows the absorption spectra of the lead halide films deposited at various molar ratios of PbBr_2_. The absorption spectrum of PbI_2_ shows an absorption onset at approximately 2.41 eV. For the sample with x = 0.1, a similar sharp peak is observed around 520 nm, which is slightly lower than that of pure PbI_2_. For the sample fabricated at the ratios of x = 0.5, the spectra are rather different. The absorption onsets for the samples at the ratios of x = 0.5 are seen at both 520 nm and 450 nm, representing the existence of the mixture of two phases, PbI_2_ and PbBr_1.2_I_0.8_. 

Because the absorbance is an additive function [35], the total absorbance, A, of the mixture of 1 and 2, where phase 1 and phase 2 have a bandgap of *Eg*_1_ and *Eg*_2_, respectively, can be expressed in terms of molar fractions as the following equation [35,36]:

(1) $$A=B_{1}\;\sqrt{\left(h\nu -E_{g1}\right)}\;f\;d+B_{2}\;\sqrt{\left(h\nu -E_{g2}\right)}\;\left(1-f\right)\;d$$

where *A* is the absorbance, *B*_1_ and *B*_2_ are constants, *f* is the fraction of the phase, and *d* is the thickness of the sample. The inset shows the Tauc plot of the sample at the ratio x = 0.5, indicating the estimated bandgaps for PbI_2_ and PbBr_1.2_I_0.8_ at 2.45 and 2.79 eV, respectively, which are in agreement with the reported values for the two phases [32].

We propose a mechanism that explains the formation of the smooth morphology of lead halide films due to the addition of PbBr_2_ during the crystallization based on the investigation of the compositional effects on the microstructure and morphology of lead halide films. For PbBr_2_ ratios up to x = 0.3, the morphology of the lead halides continues to improve, accompanied by the grain growth of PbI_2_, achieving a highly smooth surface with well-connected grains at the ratio of x = 0.3. It has been reported that in the mixed crystal Pb-I-Br, the substitution of Br for I in the solid solution generates faulted structures during the crystallization process [37,38], leading to various polytypic structures [37]. That is to say, local compositional and structural disturbance may lead to local stresses, which are relieved by creating edge (or screw) dislocations along the (001) basal planes, acting as nucleation centers for the formation of new polytypic structure [37]. This may result in many polytypes, with the dominant crystal growth taking place in the vertical direction. In addition, the spin-coated solutions consisting of PbI_2_-PbBr_2_-DMF complexes [39,40,41,42,43] undergo nucleation and growth by a post-heat treatment to complete the transformation [44]. Although such a system would be in a non-equilibrium or metastable state, the use of the quasi-binary phase diagram of PbI_2_-PbBr_2_ provides insight for the development of the microstructure through the phase transformation. For the ratio x up to x = ~0.3, only the single phase (β phase solid solution) is observed, as shown in Figure 1a, implying that approximately 30% of PbBr_2_ is dissolved in the β phase, which is much higher than the solubility limit (~5%). The rapid cooling of molten solid solution has been known to form supersaturated sold solutions with the maximum solid solubility much higher than the equilibrium value [39,45,46,47]. This can apply to the spinning process. The spin-coating of PbI_2_-PbBr_2_-DMF liquid solution may have a rapid cooling impact on the samples owing to the evaporation of solvent to produce supersaturated solid solutions with highly increased solubility. Subsequent annealing of the metastable solid solution provides a high nucleation rate and grain growth, thus resulting in the enhanced connectivity between the PbI_2_ grains.

### 3.2. Photovoltaic Performance and Long-Term Stability of Lead Halide Solar Cell

Lead halide solar cells were fabricated to investigate the effects of PbBr_2_ added to the PbI_2_ solution on device performance and stability.

**Figure 6:**
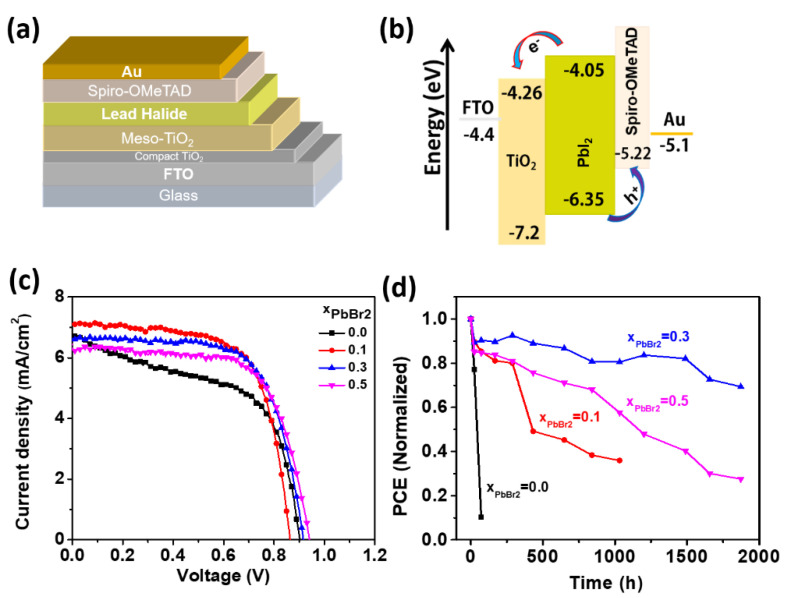
(**a**) A schematic of the device structure of the fabricated lead halide solar cell, and (**b**) the corresponding energy levels of each layer used in the device. (**c**) J-V curves of the lead halide solar cells fabricated at x = 0, 0.1, 0.3, and 0.5, respectively. (**d**) PCE of the devices fabricated at various molar ratios as a function of storage time.

Figure 6a shows a schematic of the layered structures of the fabricated lead halide solar cell. In addition, Figure 6b shows the energy level of each layer [48]. The J-V characteristics of the devices are measured to reveal the initial performance, and the degradation with storage time. Figure 6c shows the device performances obtained with the first scan, demonstrating the excellent performance of all devices, except for the device with pure PbI_2_. More specifically, the results indicate a power conversion efficiency (PCE) of 3.97–4.15% with an open circuit voltage V_oc_ of 0.86–0.94 V, a short circuit current *J_sc_* of 6.31–7.09 mA/cm^2^, and a fill factor (FF) of 67–68%, as shown in Table 1. To investigate the shelf-life stability and durability of the devices, we performed an aging test of the devices fabricated at different ratios (x). Figure 6d presents the PCE of the fabricated devices at different ratios as a function of storage time. All fabrications of the lead halide solar cells were conducted in ambient air at 28–30 °C and 25–30% humidity, and then age-tested in the same environments. The pure PbI_2_ device degraded down to almost zero within 72 h, whereas the lead halide devices demonstrated excellent stability, i.e., t_80_ = 1500 h for the device fabricated at x = 0.3, t_80_ = 500 h at x = 0.5, and t_80_ = 250 h at x = 0.1. (t_80_ implies the storage time when the PCE of the device decreases to 80% of the initial value). Such exceptional ambient stability obtained in the device fabricated at x = 0.3 can be attributed to the improved connectivity between the grains and interfaces of the β phase. The performance insensitivity of the lead halide solar cell against the environment is a major advantage for practical applications.

The P-N junction model under illumination, described as Equation (2) [49], was used to determine the origin of the performance enhancement as a result of the addition of PbBr_2_.

(2) $$J=J_{sc}-J_{0}\left[\exp\left(\frac{e\left(V+J\;R_{s}\right)}{mK_{B}T}\right)-1\right]-\frac{\left(V+J\;R_{s}\right)}{R_{sh}}$$

where *J_sc_* is the light induced current density, *J*_0_ is the reverse saturated current density of a PN heterojunction, *R_s_* is the series resistance, *R_sh_* is the shunt resistance, *J* is the current density flowing through the external load, *m* is the ideality factor of a heterojunction, *K_B_* is the Boltzmann constant, *T* is the absolute temperature, *e* is the elementary charge, and *V* is the bias applied to the cell. By differentiating, Equation (2) can be expressed as [49]

(3) $$\frac{-dV}{dJ}=mK_{B}T\left(1+R_{sh}{}^{-1}\left(\frac{dV}{dJ}\right)\right)/\left(e(J_{sc}-J-\frac{V}{R_{sh}})\right)\;+R_{s}$$

(4) $$ln\left(J_{sc}-J-\frac{V}{R_{sh}}\right)=\frac{e}{mK_{B}T}\left(V+J\;R_{s}\right)+ln\;J_{0}$$

**Figure 7:**
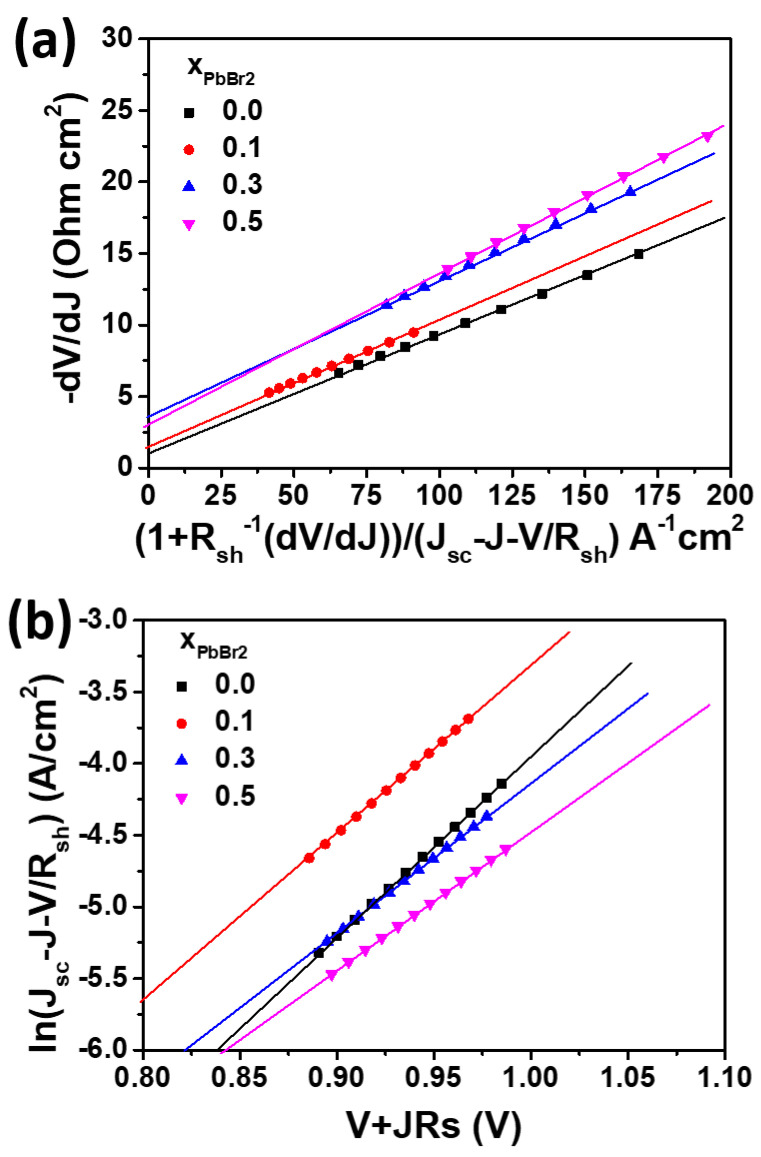
Plots of (**a**) −*dV*/*dJ* vs. (1 + *R_sh_*^−1^ (*dV*/*dJ*))/(*J_sc_* − *J* − *V*/*R_sh_*) for the solar cells fabricated at x = 0, 0.1, 0.3, 0.5 under illumination (linear fitting curves are shown), and (**b**) ln (*J_sc_* − *J* − *V*/*R_sh_*) vs. (*V* + *JR_s_*).

Figure 7a,b show the plots of −*dV*/*dJ* vs. (1 + *R_sh_*^−1^ (*dV*/*dJ*))/(*J_sc_* − *J* − *V*/*R_sh_*) and ln (*J_sc_* − *J* − *V*/*R_sh_*) vs. (*V* + *JR_s_*) and the linear fitting curves for the PbI_2(1−x)_Br_x_ solar cells according to Equations (3) and (4). From the results of the linear fitting, *m*, *R_s_*, and *J*_0_ can be calculated by the slope and intercept, respectively. The *R_sh_* is calculated from the inverse of the slope of the J-V curves near the zero bias. The measured parameters *J*_0_, *m*, *R_sh_*, and *R_s_* under illumination are shown in Table 2.

Relatively low R_s_ was obtained for all the devices. However, low shunt resistance of approximately 675 Ω cm^2^ and high ideality factor of 3.1 were obtained by the pure PbI_2_ device. Such large ideality factors (>2) imply that the carrier transport is more complex for lead iodide solar cells, compared with that for the well-behaved single heterojunction solar cell, where the ideality factor is in the range of 1 < *m* < 2. The addition of PbBr_2_ significantly increased the shunt resistance, accompanied by a slight increase in the ideality factor. Since the shunt resistance reflects the leakage current associated with bulk defects, we investigated the dark J-V curves.

**Figure 8:**
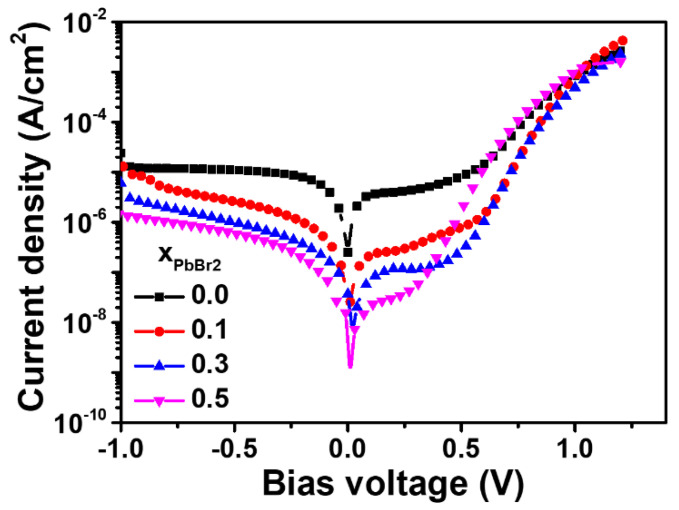
Plots of dark J-V curves for the solar cells fabricated at x = 0, 0.1, 0.3, and 0.5.

**Figure 9:**
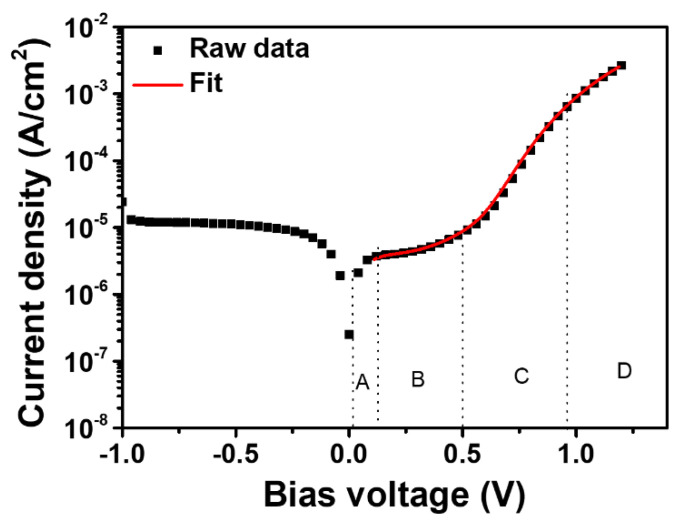
Plots of the dark current for a pure PbI_2_ solar cell as well as the fitting curve by using Equation (3). The inset regions A, B, C, and D are mainly determined by ohmic current, shunt plus capacitive current, recombination plus diffusion current in the diode region, and diode current limited by series resistance, respectively.

The dark J-V curves shown in Figure 8 were used for analysis. The leakage current significantly decreased as x increased. In addition, the dark current at low voltage is likely to consist of capacitive current and shunt current, as seen in Figure 9. Capacitive current is a result of a formed ionic capacitor from the metallic positive ions and iodide vacancies near the TiO_2_/PbI_2_ interface.

The dark current can be written as Equation (5) to include both the shunt and capacitive contributions:

(5) $$J=J_{0}\left[\exp\left(\frac{e\left(V-JR_{s}\right)}{mK_{B}T}\right)-1\right]\frac{\left(V-J\;R_{s}\right)}{R_{sh}}J_{cap}$$

where *J_cap_* is the capacitive current (=*CdV*/*dt*, *C* is the capacitance per unit area, and *dV*/*dt* is the bias scan rate).

The fitting of Equation (5) to the J-V curve was performed on the lead halide solar cell to calculate parameters such as *J_cap_* and *R_sh_*. The well fitted solid line to the J-V curve at low bias for the pure lead iodide device can be observed in Figure 9, indicating that the obtained parameters are physically meaningful.

Table 3 shows parameters, including *J*_0_, *J_cap_*, *J_sh_*, and *C*, for lead halide solar cells fabricated at various x ratios. *J*_0_ (dark) and *Jsh* are the reverse saturated current and shunt current in the dark, respectively.

At low voltages, the dark current is the sum of *J*_0_, *J_cap_*, and the shunt current. In pure PbI_2_ solar cells, the capacitive current dominates, indicating that the formed ion capacitor is the main contributor. The equation *J_cap_* = *CdV*/*dt* with *dV*/*dt* = 20 mV/s can be used to obtain the capacitance of the ionic capacitors, which results in 100 uF/cm^2^ for the PbI_2_ device [50]. Such a high capacitance can be created by the accumulation of ionic charges [51,52,53] at the contact interface which leads to a large dielectric constant of the resulting thin space charge regions near the contacts, an effect known as electrode polarization for ionic conductors [54,55,56]. For pure PbI_2_, which is known as an ionic conductor, we propose the interpretation of the capacitance as originated by the mobile ions accumulated at the mesoporous TiO_2_-PbI_2_ interface. The mobile ionic charge can be formed by the iodine anions, whose mobility can increase proportionally with the concentration of defects, such as iodine vacancies, grain boundaries, and interlayer voids. As the PbBr_2_ ratio increases, the content of iodine vacancies decreases, as shown in Figure 4, and the capacitive current decreases accordingly. As a result, the addition of PbBr_2_ significantly decreases the defects, such as vacancies, grain boundaries, and voids, and thus leads to enhanced electrical performance and stability. 

**Figure 10:**
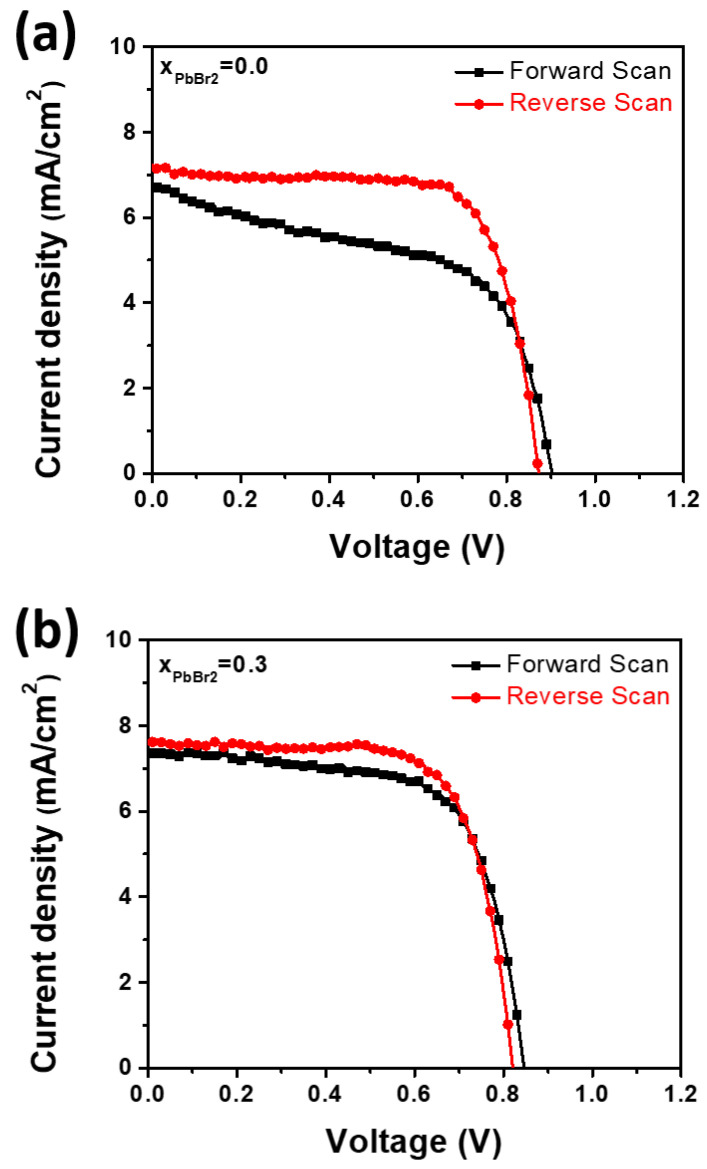
J-V curves measured by forward (from *J_sc_* to *V_oc_*) and reverse (from *V_oc_* to *J_sc_*) scans for the solar cells fabricated at x = (**a**) 0.0 and (**b**) 0.3.

In addition to a long shelf-life, the hysteresis-free electrical characteristics are desirable for photovoltaic devices. Figure 10 presents the J-V curves for solar cells fabricated with pure PbI_2_ and the mixture of PbI_2_ and PbBr_2_ solutions, measured in forward (that is, from *J_sc_* to *V_oc_*) and reverse (from *V_oc_* to *J_sc_*) modes under standard air mass 1.5 global (AM 1.5 G) illumination. It can be observed that there is a large hysteresis in the J-V curve of the pure PbI_2_ based solar cell, showing a pronounced discrepancy of approximately 10% in overall efficiency. In contrast, the J-V curves of the forward and reverse scans of the mixture-based cells were relatively coincident with a decreased discrepancy of approximately 2% in the overall efficiency. The improvement in hysteresis can be due to the reduction in mobile ions such as iodine ions, probably resulting from the reduction in the structural defects, including iodine vacancies, grain boundaries, and dislocations.

## 4. Conclusions

In this paper, we investigate the compositional effects of the molar ratio (x) of PbBr2 on the crystal structure, preferred orientation, optical property, microstructure, surface morphology, and defects of the mixed lead halides PbI_2(1__−x)_Br_2x_ formed on meso-TiO_2_ substrates. For the ratio x ≤ 0.3, the addition of PbBr_2_ has a remarkable effect on the morphology and microstructure of lead halide films. As PbBr_2_ increases, the size of the (001)-oriented PbI_2_ grains increases, and the surface morphology significantly improves, indicating that the growth of the PbI_2_ grains dominates and is responsible for the continuous and uniform surface of the lead halides films coated with a molar ratio x = 0.3. The mechanism for the growth of the Br-supersaturated β phase from the spin-coated PbI_2_–PbBr_2_-DMF complexes is proposed by considering the quasi-binary phase diagram of PbI_2_-PbBr_2_. The use of the phase diagram provided an insight regarding the control of the microstructure of thin films, which can be applicable to the development of metal-halide perovskite films. In addition, XPS analysis confirmed the anion vacancies associated with metal Pb in the lead halide films, which decreased by the addition of PbBr_2_. At x = 0.5, discontinuous lead halides consisting of the two phases, of β phase plus PbBr_1.2_I_0.8_, were formed, producing a rough surface. Measurement of the optical absorbance of the lead halide films fabricated at x = 0.5 shows the two absorption edges for saturated PbI_2_ and PbBr_1.2_I_0.8_, indicating that the energy bandgap of PbI_2_ and PbBr_1.2_I_0.8_ is 2.48 and 2.79 eV, respectively. 

Finally, we fabricated lead halide-based solar cells at various ratios of PbBr_2_ to PbI_2_. The solar cell produced at x = 0.3 exhibited excellent device performance with long-term stability and without any encapsulation. The enhanced performance with long-term stability is due to the reduction in structural defects by the addition of PbBr_2_. These results demonstrate the device quality of lead halide films produced from mixed lead halides, offering their potential feasibility for wide bandgap device applications, including semitransparent/transparent and tandem solar cells.

## Figures and Tables

**Table 1:** Photovoltaic performance parameters of the lead halide solar cell at different molar ratio of PbBr_2_.

x	*J_sc_* (mA/cm^2^)	*V_oc_* (V)	FF	PCE (%)
0.0	6.74	0.90	0.55	3.36
0.1	7.09	0.86	0.68	4.15
0.3	6.63	0.92	0.67	4.11
0.5	6.31	0.94	0.67	3.97

**Table 2:** The measured parameters such as *J*_0_, *m*, *R_sh_*, and *R_s_* for the devices fabricated at various x under illumination.

	x = 0.0	x = 0.1	x = 0.3	x = 0.5
*J*_0_	7.1 × 10^−8^	2.6 × 10^−7^	4.1 × 10^−7^	6.9 × 10^−7^
*m*	3.1	3.2	3.6	3.9
*R_sh_*	675	4.74 × 10^4^	2.75 × 10^4^	2.04 × 10^6^
*R_s_*	1.42	1.79	3.76	3.47

**Table 3:** The measured parameters from the dark J-V curves of lead halide devices.

	x = 0.0	x = 0.1	x = 0.3	x = 0.5
*J*_0_ (dark) (A/cm^2^)	1.9 × 10^−9^	1.0 × 10^−11^	1.0 × 10^−11^	3.7 × 10^−10^
*J_cap_* (A/cm^2^)	2 × 10^−6^	1 × 10^−7^	8 × 10^−8^	2 × 10^−8^
*C* (μF/cm^2^)	100	5	4	1
*R_sh_* (dark) (Ω cm^2^)	2 × 10^5^	9 × 10^5^	4 × 10^6^	5 × 10^6^
*J_sh_* (=*V*/*R_sh_*) at 0.1 V	5 × 10^−7^	1.1 × 10^−7^	2.5 × 10^−8^	2 × 10^−8^

## Data Availability

Data are available on request.
